# Supplementary material for: Complexome profiling of the Chlamydomonas psb28 mutant reveals TEF5 as an early PSII assembly factor
Source: Plant Cell. 2025 Mar 17;37(6):koaf055. doi: 10.1093/plcell/koaf055 (PMC12164590; doi:10.1093/plcell/koaf055)
Supplement: koaf055_Supplementary_Data [file koaf055_supplementary_data.zip › psb28_interactive.zip!/plots/intensity/qualityPlots/peptides/PGK1_Cre11.g467770.t1.1.html]

### Cre11.g467770.t1.1

Trivial name: PGK1  
SeqCoverage [median]: 27.30  
Mass: 49.03 kDa  
  
 

| Sequence | MissedCleavage | Mass [Da] | wtIntensity | muIntensity | Score |
| --- | --- | --- | --- | --- | --- |
| AAVPTLK | 0 | 698.43 | 0.00 | 617.93 | 77.75 |
| ADLNVPLDK | 0 | 983.53 | 960.01 | 1675.08 | 101.65 |
| ADLNVPLDKATLAITDDTR | 1 | 2041.07 | 0.00 | 1902.10 | 45.61 |
| ATLAITDDTR | 0 | 1075.55 | 789.85 | 0.00 | 102.07 |
| ELDYLDGAVSNPK | 0 | 1419.69 | 847.39 | 1106.59 | 143.50 |
| FYKEEEKNEPEFAK | 2 | 1786.84 | 0.00 | 393.19 | 26.12 |
| LAANADLYVNDAFGTAHR | 0 | 1917.93 | 0.00 | 1329.52 | 63.92 |
| MSHISTGGGASLELLEGK | 0 | 1785.89 | 0.00 | 1085.77 | 68.58 |
| RPFVAIVGGSK | 1 | 1129.66 | 0.00 | 883.50 | 76.06 |
| VGSSLVEDDKIELAK | 1 | 1601.85 | 0.00 | 4093.57 | 60.20 |
| VLPGVAALDEK | 0 | 1110.63 | 0.00 | 1775.46 | 57.55 |
|  |  |  | 2597.2 | 14862.7 |  |
